# Impact of endoscopic enucleation of the prostate with thulium fiber laser on the erectile function

**DOI:** 10.1186/s12894-018-0400-1

**Published:** 2018-10-12

**Authors:** Dmitry Enikeev, Petr Glybochko, Leonid Rapoport, Zhamshid Okhunov, Mitchel O’Leary, Natalya Potoldykova, Roman Sukhanov, Mikhail Enikeev, Ekaterina Laukhtina, Mark Taratkin

**Affiliations:** 1 Institute for Urology and Reproductive Health, Sechenov University, Moscow, Russia; 2 Department of Urology, University of California, Irvine; Orange, CA USA

**Keywords:** Thulium fiber laser, Endoscopic enucleation of the prostate, BPH, Erectile function, ThuFLEP

## Abstract

**Background:**

The impact of number of endoscopic enucleation of the prostate techniques (holmium laser enucleation - HoLEP for example) on erectile function have already been investigated. However, the thulium-fiber laser, in this setting remains unstudied. In this study, we compared sexual function outcomes in patients with benign prostatic hyperplasia (BPH) treated with transurethral resection of the prostate (TURP) or thulium-fiber laser enucleation (ThuFLEP).

**Methods:**

We performed a retrospective analysis of patients who underwent transurethral resection and endoscopic enucleation of the prostate for BPH; inclusion criteria was the presence of infravesical obstruction (IPSS > 20, Qmax < 10 mL/s). Erectile function (EF) was assessed using the International Index of Erectile Function (IIEF-5) both prior to endoscopic examination, and six months after.

**Results:**

A total of 469 patients with BPH were included in the study; of these, 211 underwent to ThuFLEP, and 258 TURP. Preoperative IIEF-5 in TURP and ThuFLEP groups were 11.7 (±4.5) and 11.1 (±5.0), respectively (*p* = 0.17). At six month the IIEF-5 score was unchanged (*p* = 0.26 and *p* = 0.08) and comparable in both groups (*p* = 0.49). However, mean IIEF-5 score shown significant increase of 0.72 in ThuFLEP group, comparing to decrease of 0.24 in TURP patients (*p* < 0.001).

**Conclusions:**

Both TURP and ThuFLEP are effective modalities in the management of infravesical obstruction due to BPH. At six months follow-up after surgery, both techniques lead to comparable IIEF-5 score. However, our results demonstrated that the ThuFLEP is more likely to preserve the erectile function leading to increase of IIEF-5 at six months in contrast to TURP which lead to slight drop in IIEF-5 score.

## Background

Benign prostatic hyperplasia (BPH) is expected to afflict 50% of men over the age of 50 [1]. It has been demonstrated [2–4] that there is an association between BPH and erectile dysfunction (ED). BPH has also been shown to deteriorate the existing erectile disturbances or to become one of the causes of its development [2]. Conversely, timely surgical treatment of BPH (i.e. transurethral resection of the prostate (TURP)) has been shown to perturb the development of erectile dysfunction [4]. As the BPH in most cases is not a life-threatening condition, the main outcomes of it is treatment not only the improvement in an international prostate symptom score (IPSS) (as the outcome that shows the micturition quality), but the men’s quality of life after surgery. With significant sexual activity of aging males the question of the effect of transurethral surgery on erectile function is prominent [5].

The impact of number of endoscopic enucleation of the prostate techniques on erectile function has already been investigated [6, 7]. A study assessing the influence of a thulium fiber laser (Tm-fiber) enucleation of the prostate (ThuFLEP), however, is currently lacking. Different from widely used Tm:YAG laser (in ThuLEP and ThuVEP techniques), Tm-fiber laser allows to minimize penetration depth (2-times in comparison to Tm:YAG), which reduces tissue damage and allows to perform precise incisions [8]. This is possible due to Tm-fiber laser wavelength of 1940 nm (vs 2010 nm of Tm:YAG), which leads to increase of laser energy absorption in tissue, allowing to decrease the penetration depth and leading to instantaneous vaporization [9, 10]. Also, it is believed that the use of Tm-fiber laser may to decrease the carbonization rate, comparing to Tm:YAG lasers [9]. Unlike the Tm:YAG laser which consists of several flash-lamp pumped Tm:YAG crystals the Tm-fiber laser use in it is construction the laser fiber pumped by diode laser, which leads to difference in wavelength and smaller size of the laser device.

The objective of this study was to assess the efficacy and safety of the Tm-fiber laser and evaluate its impact on erectile dysfunction (ED) in patients who underwent ThuFLEP comparing to patients, who underwent the standard monopolar TURP.

## Methods

### Patient identification and data collection

We performed a retrospective analysis of patients who underwent ThuFLEP or TURP for infravesical obstruction due to BPH between December 2012 and February 2018. Inclusion criteria was the presence of infravesical obstruction (defined as IPSS > 20, Qmax < 10 mL/s). Patients were excluded from analysis if they had prostate cancer, urethral strictures, or bladder calculi.

### Surgical technique

We used the Urolase (NTO IRE-Polus, Russia) (120 W) Tm-fiber laser with wavelength of 1940 nm and a 600-μm laser fiber, and a 26 Ch resectoscope (Karl Storz, Germany) with continuous irrigation (0.9% saline). All ThuFLEP procedures were performed as previously described [11]. In ThuFLEP technique (Tm fiber laser) we usually perform 70% of the surgery dissecting the tissue with laser energy and only 30% of the surgery are usually done in blunt enucleation, in contrast to ThuLEP technique (Tm:YAG laser) with exact opposite ratio of blunt and laser dissection.

Instantaneous vaporization of the tissue and small penetration depth of Tm-fiber laser allows to perform fast and precise incisions with minimal need for coagulation of bleeding vessels. All procedure steps, except the incision at veromontanum was performed in 60 W (1.5 J) power setting, however at veromontanum we decreased the energy to 30 W. We suggest that it may preserve the sphincter zone and decrease the incontinence rate. Adenomatous tissue was retrieved using a 5-mm cystoscope and a morcellator (Piranha, Richard Wolf, Germany). Monopolar TURP (5% glucose) with Ch 24 resectoscope (Karl Storz, Germany) was performed on 258 patients whose ages varied between 54 and 83 years (average 68.0 ± 6.7 years). Prostatic vessels were coagulated with a roller electrode (if necessary).

### Study outcomes

Primary outcome of the study was to assess the difference in erectile function (EF), which was measured using the five-item version of the International Index of Erectile Function (IIEF-5) both prior to surgery and six months after. Secondary outcome of the study was decrease of infravesical obstruction severity, which was assessed using International Prostate Symptom Score (IPSS) and the maximum flow rate (Qmax).

### Statistical analysis

Baseline characteristics, perioperative data, and descriptive statistics from the procedures were collected. Continuous variables were compared by one-way ANOVA test. Categorical variables were compared by via Chi square tests. Nonparametric variables were compared with Kolmogorov–Smirnov and Kruskall-Wallis tests. Post Hoc analysis was performed with Mann-Whitney U Test. Propensity score matching for comparison of patients with different prostate volume and surgery time was done (with SPSS Propensity score matching). All statistical analyses were carried out using SPSS Statistics 23.0 (SPSS Inc., Chicago, IL, USA). A *p*-value < 0.05 was considered to indicate statistical significance.

## Results

A total of 469 patients were included in the study. The mean age of the patients subjected to ThuFLEP (211) was 67 ± 7.4 years. The average prostate volume in this group was 90 ± 42.9 cc (30–250 cc), with an average PSA of 4.7 ± 2.7 ng/mL. The mean age in TURP group (258) was 68 ± 6.7 average prostate volume in TURP group was 63.0 ± 17.1 cc (30–89 cc), with a total PSA level of 4.2 ± 2.3 ng/mL (Table 1). The larger prostate volume in ThuFLEP group was not suggested as the limitation, because both groups were comparable in preoperative IPSS, Qmax and IIEF-5 scores (*p* = 0.22; *p* = 0.06; *p* = 0.17) (Table 2) (additional analyses with propensity score matching was done).

**Table 1:** Patients demographics and clinical characteristics

	ThuFLEP (*n* = 211)	TURP (*n* = 258)	*p*
Age (years, mean, range)	67 ± 7.4	68 ± 6.7	0.22
Prostate volume (cc, mean, range)	90 ± 42.9	63 ± 17.1	< 0.001*
PSA (ng/ml, mean, range)	4.7 ± 2.7	4.2 ± 2.3	0.03*

*statistically significant difference. Data given as mean ± SD

**Table 2:** Pre- and postoperative functional results at six months

	ThuFLEP (*n* = 211)	TURP (*n* = 258)	*p*
IPSS – preop. (score)	21.8 (± 1.6)	21.6 (±1.7)	0.22
IPSS – postop. (score)	10.9 (± 3.0)	10.6 (±3.2)	0.35
*p*	< 0.001*	< 0.001*	
QoL – preop. (score)	4.0 (± 0.8)	3.9 (±0.8)	0.23
QoL – postop. (score)	1.8 (± 0.6)	1.7 (±0.6)	0.38
*p*	< 0.001*	< 0.001*	
Qmax – preop. (ml/s)	7.5 (± 1.7)	7.8 (±1.9)	0.06
Qmax – postop. (ml/s)	16.2 (± 3.3)	16.6 (±1.5)	0.08
*p*	< 0.001*	< 0.001*	
PVR – preop. (ml)	70.1 (± 28.7)	68.7 (±21.5)	0.08
PVR – postop. (ml)	17.3 (± 11.7)	15.3 (±13.6)	0.08
*p*	< 0.001*	< 0.001*	
IIEF-5 – preop. (score)	11.1 ± 5.0	11.7 ± 4.5	0.17
IIEF-5 – postop.(score)	11.7 ± 4.7	11.5 ± 4.7	0.49
*p*	0.08	0.26	
IIEF-5 change	▲0.72 ± 1.6	▼0.24 ± 2.2	*p* < 0.001*

*statistically significant difference; ▲ – increase of score; ▼– decrease of score. Data given as mean ± SD

The average operative time was 72 min in the ThuFLEP group, and 54 min in the TURP group. A urethral catheter was left in place for 1–2 days in the ThuFLEP group, and 3–4 days in the TURP group. Average hospital stay was 3 and 5 days for the ThuFLEP and TURP groups, respectively. The ThuFLEP duration was longer than TURP (*p* < 0,001) (due to larger prostate volume and technical aspects of techniques). The catheterization length and hospital stay were in favor of ThuFLEP (*p* < 0,001). At six months follow up, each group had a significant improvement in the IPSS, QoL and Qmax (Table 2).

IIEF-5 score in TURP group remained stable (average preoperative value: 11.7 ± 4.5; average postoperative value: 11.5 ± 4.7). EF following TURP was unchanged in 43% of patients; improved EF - 21% of patients and impaired EF in 34% of patients. De novo erectile dysfunction (mild) was found in 5 (2%) patients.

**Fig. 1:**
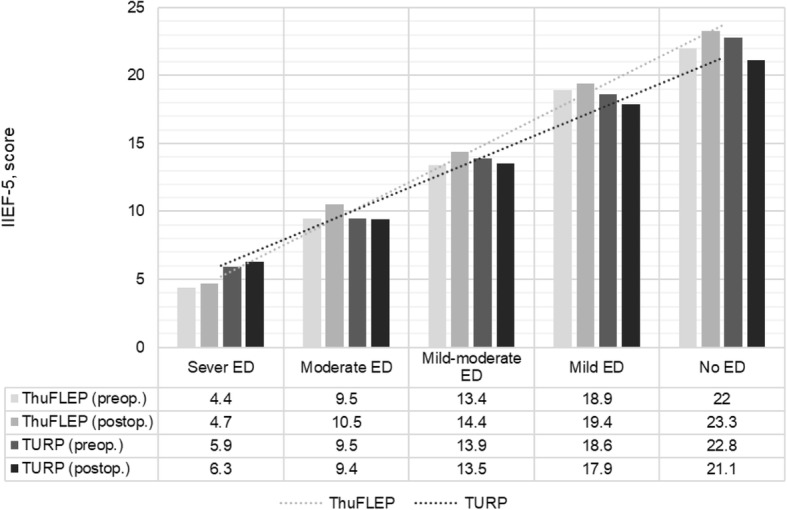
IIEF-5 prior and six months after the surgery

Similarly, no significant change in EF was seen in patients subjected to ThuFLEP. The average IIEF-5 value before surgery was 11.1 ± 5.0, and that 6 months after surgery was 11.7 ± 4.7. The EF following ThuFLEP remained stable in 56%, improve in 26% of patients and decreased in 18% of patients. There was no de novo ED in patients within the ThuFLEP group. Therefore, both techniques, were comparable in postoperative IIEF-5. However, mean increase of IIEF-5 score in ThuFLEP group was about 0.72 ± 1.6 while the IIEF-5 score in TURP group show decrease of 0.24 ± 2.2 (Table 2). This difference between the two techniques was statistically significant (*p* < 0.001) (Fig. 1). Due to difference in the preoperative prostate volume and operative time we did propensity score matching, which has confirmed our results with mean decrease of IIEF-5 in TURP patients of 0.24 and increase in ThuFLEP group of 0.7 (*p* < 0.001), no difference were found in pre- and postoperative IIEF-5 scores between two groups (*p* = 0.09 and *p* = 0.77, respectively).

**Fig. 2:**
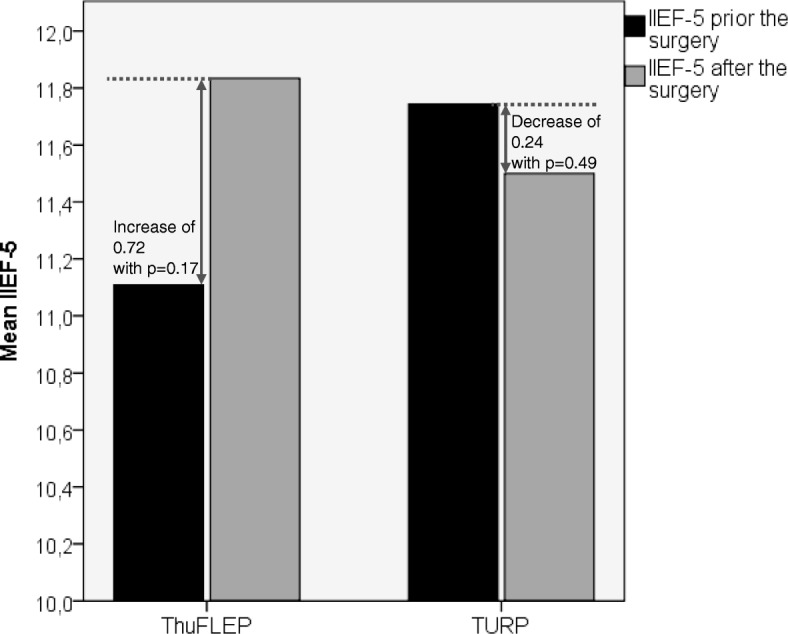
IIEF-5 score in patients with different stages of ED

Our next step was to compare the erectile function change in groups of patients with different stages of ED (assessed with IIEF-5 score) (Fig. 2). In patients with severe ED (1–7) no difference between TURP and ThuFLEP (*p* = 0.76) was observed, moreover both techniques allowed slight, yet significant increase of EF (*p* < 0.001). In moderate ED group (8–11) ThuFLEP showed no influence on EF, whereas TURP led to slight decrease (*p* = 0.05), with significant difference between the techniques (*p* = 0.002). In patients with mild-moderate ED (12–16) ThuFLEP led to increase (*p* = 0.04) and TURP to decrease of EF (*p* = 0.031) with significant difference between the techniques (*p* = 0.001). In patients with mild ED (12–16) or without ED (22–25) ThuFLEP showed no influence on IIEF-5 score (*p* = 0.617 and *p* = 0.192, respectively), whereas TURP decreased IIEF-5 score in patients with mild ED (p = 0.04) and had no influence on the patients without ED (*p* = 0.08).

To estimate the possible influence of other EF-affecting diseases and disorders we compared the IIEF-5 differences in patients with obesity (ThuFLEP – 55 and TURP – 68), cardiovascular diseases (ThuFLEP 40 and TURP 65) and diabetes mellitus (ThuFLEP 15 and TURP 17). We did not observe increase or decrease of EF for such patients in both in ThuFLEP and TURP groups (*p* = 0.1, *p* = 0.1 and *p* = 0.257, respectively).

Among short-term complications most frequent was clot retention, which was found in 17 (6.6%) and 9 (4.3%) patients after TURP and ThuFLEP, respectively (0.373). In three patients (1.4%) after ThuFLEP we encounter superficial bladder wall damage with the morcellator. One patient after TURP necessitated blood transfusion (0.4%), and one had TURP-syndrome (0.4%). Six months after surgery 10 (3.9%) patients in TURP group and 2 (0.9%) in ThuFLEP group had urinary incontinence (*p* = 0.9); urethral stricture was found in 3 (1.2%) after TURP and 1 (0.5%) after ThuFLEP (0.806), the bladder neck sclerosis was found in 5 (1.9) and 1 (0.5) patient (*p* = 0.088) after TURP and ThuFLEP, respectively.

## Discussion

At six months, both surgical modalities were equally efficacious in eliminating infravesical obstruction due to BPH. A longer thulium enucleation time demonstrated in our study (72 min versus 54 min) is mostly attributed to a larger BPH volume within the ThuFLEP group (90 cc on average) compared to the TURP group (BPH is 63 cc on average). However, propensity score matching allowed to rule out it is influence on the results.

The results of recent meta-analyses and systematic reviews show endoscopic enucleation to be highly efficacious in the treatment of BPH, and to have comparable postoperative outcomes to TURP [12–14]. Both techniques are very effective regarding IPSS and Qmax outcomes, however, the effect of surgical treatment of benign prostatic enlargement (BPE) on EF after surgery remain the subject of discussion amongst Urologists. Certain authors [15] suggest that ED is function of age; others relate it to preexisting ED [16]. Hanbury et al. [17] suggest that erectile dysfunction may be caused by intraoperative injury of the prostatic capsule and the adjacent neurovascular bundles during TURP. According to the data of different investigations [15, 18, 19], ED as a consequence of TURP occurs in up to 35% of patients. Nevertheless, it should be mentioned that certain decrease of EF in these patients is frequently diagnosed before surgical intervention [15].

In our work, the erectile function following TURP remained intact in 43% of cases. EF was restored in 21% of patients, and impaired in 36% of patients. No statistically significant differences between the pre- and postoperative means were noted according to IIEF-5 score assessment. These facts may indicate that the EF did not change significantly after TURP. Similar data was obtained by Muenter et al. [20]. They note that TURP did not lead to changes in EF within 52% of patients, and that EF improved, albeit insignificantly, in 29%. Moreover, EF was shown to decrease in only 19% of their patients. The authors believed, however, that the reason for such decrease is the neurovascular bundles damage, because of the generated monopolar current passed in close proximity to the prostatic capsule [20]. This theory was in part confirmed by Li et al. [21], as they indicated in their meta-analysis a predominantly small EF decrease during a short-term follow-up. However, at a follow-up of 12 months after TURP, the average EF values returned to normal and did not differ from those recorded preoperatively, especially in patients presenting with an initially high EF value [21].

In turn, the holmium and thulium lasers are distinguished by a smaller tissue penetration depth when compared to the electrocoagulation used in TURP [10]. According to EAU guidelines on laser technologies [10], the application of thulium laser enhances hemostasis, minimizes the degree of damage to the underlying tissues, and allows the capsule and the neurovascular bundles intimately adjacent to the posterolateral surface of the prostate to remain intact. Tiburtius et al. [22] demonstrated the action of a thulium:YAG laser on the erectile function, and found a small but significant increase (from 19 to 20) in the IIEF-5 score at a follow-up of 12 months. This finding was attributed to the shallow penetration depth of Tm:YAG energy.

According to our data, the average outcomes as assessed by IIEF-5 before and after surgery did not change substantially. However, it was shown that in contrast to TURP, ThuFLEP allows to significantly increase IIEF-5 score. Fried et al. suggested that the shallow laser penetration depth of a Tm-fiber laser is due to its wavelength being close to that of the water absorption peak [9]. Prostate tissues contain a considerable amount of water, and the energy is transmitted to tissues more effectively at such a wavelength [9, 10]. Thus, safer incisions can be made at a lower risk of perforating the surgical capsule and damaging the neurovascular bundles intimately adjacent to the posterolateral surface of the prostate. Another possible explanation may be that enucleation procedure itself allows significant increase of urinary function and faster rehabilitation, which together with increase of quality of life may facilitate EF recovery.

This suggestion for thulium laser is supported by Iacono et al. [23]: at 12 months after surgery, the erectile function was completely restored in all the patients enrolled in the study, a finding linked to the low probability of perforation of the surgical capsule found with employing a thulium laser. This reduces the risk of erectile dysfunction by means of an injury to the neurovascular bundles. Similar data was obtained by Chung et al. [24] who described EF to decline three months after ThuLEP performance. ED was revealed in both patients with already existing erectile disturbances, and in those without erectile complaints. Close correlation was observed between the IIEF-5 score assessment and the age of the patients. It was noted that the older the patient, the more marked the decline in EF was. At 12 months of follow-up, however, the functional outcomes as assessed by the IIEF-5 returned to the preoperative level in both groups. This led them to conclude that ThuLEP does not have a long-term negative impact on erectile function [24]. However Iacono et al. and Chung et al. studies were conducted with Tm:YAG laser, which is different from Tm-fiber laser technology and ThuFLEP procedure [23, 24].

In our study, the erectile function after ThuFLEP remained unchanged in 56% of patients. The functional outcomes as assessed by the IIEF-5 score after surgery improved in 26% of patients and decreased in 18% of observations. In most patients, no changes in the erectile function were noted. As for the patients with obesity, cardiovascular diseases and diabetes mellitus, no change in EF was noted, which may signify that in such patients EF was affected not with urination disorders/BPH, but with other pathology. In groups of the patients with different stages of preoperative ED (from “sever ED” to “No ED”) we found that ThuFLEP allowed to preserve EF in all groups and even increase it in patients with severe and mild-moderate ED. TURP led to increase of EF in patients with severe ED, showed no influence on the patients without ED and decreased EF in all other groups. Generally, the IIEF-5 decrease in TURP by 0.24 and increased by 0.72 in ThuFLEP group. Such a slight difference between two techniques, may not be of high clinical significance, still it shows that ThuFLEP is more likely to increase or preserve EF in contrast to TURP. Which in turn mean, that laser enucleation with Tm-fiber laser may be considered as one of the possible techniques of choice for patients who concerned of theirs EF. Still, this statement is theoretical, and further investigation is necessary.

Among main limitations of the study were its retrospective nature and absence of long-term data (up to 12 months). Another limitation was use of the simplified IIEF version – IIEF-5 questionnaire, which did not allow us to precisely estimate changes in the different components of erectile function.

## Conclusions

Both TURP and ThuFLEP have shown to be effective in the management of infravesical obstruction due to BPH. Despite the absence of statistically significant differences in the IIEF-5 assessments before and after surgery, the application of a Tm-fiber laser in 26% of patients with significant ED resulted in the improvement in EF. In contrast to TURP it allowed to perform slight, but significant increase of IIEF-5. ThuFLEP can be considered to retain, and in certain cases, increase the erectile function.

## Abbreviations

BPH: Benign prostatic hyperplasia
ED: Erectile dysfunction
EF: Erectile function
HoLEP: Holmium laser enucleation
IIEF-5: International Index of Erectile Function
IPSS: International prostate symptom score
QoL: Quality of life
ThuFLEP: Tm-fiber laser enucleation
ThuLEP: Thulium laser enucleation
Tm-fiber: Thulium fiber laser
TURP: Transurethral resection of the prostate
